# Proteomics Analysis of the Spinal Dorsal Horn in Diabetic Painful Neuropathy Rats With Electroacupuncture Treatment

**DOI:** 10.3389/fendo.2021.608183

**Published:** 2021-06-10

**Authors:** Xiangmei Yu, Xiaomei Chen, Weiting Liu, Menghong Jiang, Zhifu Wang, Jing Tao

**Affiliations:** ^1^ College of Integrated Traditional Chinese and Western Medicine, Fujian University of Traditional Chinese Medicine, Fuzhou, China; ^2^ College of Rehabilitation Medicine, Fujian University of Traditional Chinese Medicine, Fuzhou, China; ^3^ College of Acupuncture and Moxibustion, Fujian University of Traditional Chinese Medicine, Fuzhou, China

**Keywords:** proteomics, electroacupuncture, oxidative phosphorylation, neuropathic pain, diabetes

## Abstract

**Background:**

Clinical evidence demonstrates that electro-acupuncture (EA) of the Zu sanli (ST36) and Shen shu (BL23) acupoints is effective in relieving diabetic painful neuropathy (DPN); however, the underlying molecular mechanism requires further investigation, including the protein molecules associated with EA’s effects on DPN.

**Methods:**

Sprague-Dawley adult male rats (n =36) were randomly assigned into control, DPN, and EA groups (n=12 each). After four weeks of EA treatment, response to mechanical pain and fasting blood glucose were analyzed. A tandem mass tag (TMT) labeling approach coupled with liquid chromatography with tandem mass spectrometry was used to identify potential biomarkers in the spinal dorsal horn. Further, proteomics analysis was used to quantify differentially expressed proteins (DEPs), and gene ontology, KEGG pathways, cluster, and string protein network interaction analyses conducted to explore the main protein targets of EA.

**Results:**

Compared with the DPN model group, the mechanical pain threshold was significantly increased, while the fasting blood glucose levels were clearly decreased in EA group rats. Proteomics analysis was used to quantify 5393 proteins, and DEPs were chosen for further analyses, based on a threshold of 1.2-fold difference in expression level (P < 0.05) compared with control groups. Relative to the control group, 169 down-regulated and 474 up-regulated proteins were identified in the DPN group, while 107 and 328 proteins were up- and down-regulated in the EA treatment group compared with the DPN group. Bioinformatics analysis suggested that levels of proteins involved in oxidative stress injury regulation were dramatically altered during the EA effects on DPN.

**Conclusions:**

Our results provide the valuable protein biomarkers, which facilitates unique mechanistic insights into the DPN pathogenesis and EA analgesic, antioxidant stress and hypoglycemic effect.

## Introduction

There are various types of chronic pain that can disturb the physical and mental health of individuals. Diabetic painful neuropathy (DPN) is a painful complication of diabetes, caused by constant high glucose levels. Approximately 20% of all diabetic patients present with neuropathic pain, manifesting as spontaneous pain, hyperalgesia, and allodynia (1). The streptozotocin (STZ)-diabetic rat model can be used to mimic the symptoms and pathological changes that occur during DPN. To date, the majority of studies have focused on peripheral sensory nerve function; however, the spinal cord is also important in the development of DPN (2). A number of morphological studies have demonstrated spinal cord damage and atrophy in patients with diabetes, and peripheral nerve stimulation can reduce blood-oxygen level-dependent activity in the dorsal horn of diabetic rats (3, 4).

Electroacupuncture (EA), as a beneficial acupuncture therapy, is effective in alleviating pain and improving quality of life in patients with diabetic peripheral neuropathy (5–7). Zusanli (ST36) and Shenshu (BL23) are common and effective acupoints used for treatment of diabetic peripheral neuropathy (8–10).

Proteomics analysis provides a valuable strategy for exploring the pathogenesis of diabetes mellitus, as well as therapeutic targets in this condition. Some proteomic studies have been performed in animal models of DPN or diabetic neuropathy, to search for differentially expressed proteins (DEPs) induced by STZ. Mitochondrial oxidative phosphorylation, metabolic dysregulation of mitochondrial complexes, and antioxidases are involved in DPN progression and represent potentially important therapeutic targets. However, previous studies have focused only on the proteomes of the sciatic nerve, lumbar 4/5 dorsal root ganglia, and trigeminal ganglia, while the exact spinal mechanism underlying DPN is far from clear and additional potential spinal biomarkers are needed (11–14).

## Materials and Methods

### Animals and Ethics Statement

Male Sprague-Dawley rats weighing 160 ± 20g were obtained from the Experimental Animal Center of Fujian University of Chinese Medicine. Rats were housed under standard living conditions at 22°C and a 12 h light/dark cycle with sufficient food and water and acclimatized to the pain-testing environment before the experiments. All animal procedures were in strict accordance with relevant international laboratory animal use guidelines. After adaptive feeding, rats were randomly allocated into three groups as follows: Control group (n=12), DPN group (n=12), and EA group (n = 12).

According to the previous literature reports, diabetes was induced by a single intraperitoneal injection of STZ (65 mg/kg, Sigma Chemicals, USA) dissolved in citrate buffer (10 mmol/L, Na citrate, pH=4.3) (15). The control group received an equivalent volume of citrate buffer only. Two weeks later, tail venous fasting blood glucose (FBG) were measured using a glucometer (OEM, USA). Only rats with FBG >16.7 mmol/L and mechanical paw withdrawal threshold (PWT) <5 g were included in further experiments.

All experimental procedures and protocols were approved by the Animal Research and Ethics Committee of Fujian University of Traditional Chinese Medicine. At the endpoint of the experiment, all rats were euthanized, according to the Care Guidelines.

### Electroacupuncture (EA) Procedure

**Figure 1:**
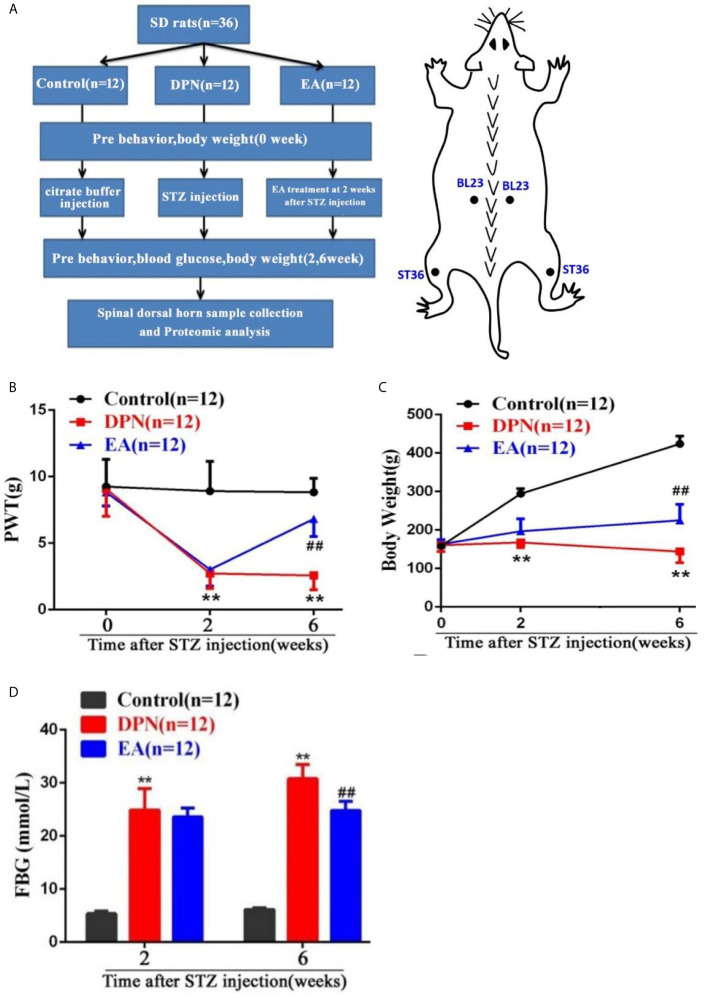
Flow chart of the experiment and the changes of body weight, paw withdrawal threshold (PWT) and fast blood glucose (FBG) in DPN rats. **(A)** Flow chart of the experiment. **(B)** Body weight variation in Control, DPN, and EA groups rats. Two-way repeated ANOVA, F2, 33 = 237.4, P < 0.001; *post hoc* Turkey test: DPN compared with Control (**P < 0.01), EA compared with DPN (^##^ *P* < 0.01). **(C)** Significant changes of PWT in different groups. Two-way repeated ANOVA, F2, 33 = 59.608, P < 0.001; *post hoc* Turkey test: DPN compared with Control (**P < 0.01), EA compared with DPN (^##^ *P* < 0.01). **(D)** Significant changes of FBG in different groups. Two-way repeated ANOVA, F1, 33 = 299.3, P < 0.001; *post hoc* Turkey test: DPN compared with Control (**P < 0.01), EA compared with DPN (^##^ *P* < 0.01).

In the EA group, the rats were loosely fixed on a wooden stand, where their head and limbs could move freely. After the rats were stabilized, the acupoints “Zu sanli” (ST36) and “Shen shu” (BL23) were selected for acupuncture and electrical stimulation (**Figure 1A**). EA intervention began at two weeks after STZ injection. ST36 is located at the posterolateral knee about 5mm below the fibula head (16), while BL23 is located in the depressions lateral to the lower border of the spinous processes of the second lumbar vertebrae, approximately 8mm from the midline of the adult rat (17). Needles inserted at ST36 and BL23 were connected to a G6805-1A multifunctional EA apparatus (Shanghai Medical Electronic Apparatus Company, Shanghai, China), with a stimulation intensity of 1mA, frequency 10 HZ. According to previous literature reports, EA stimulation was maintained for 30 min once every other day for a consecutive four weeks (15, 18).

### Measurement of Paw Withdrawal Threshold

The mechanical PWT was examined before and after injection of STZ or citrate buffer (0, 2, 6 weeks after injection). The test environment was a 10 cm × 20 cm × 20 cm plastic cage with a plexiglass floor. After the rats were placed in the cage and allowed to stand for 10 minutes, the center of the feet of the rats were stimulated with different tense by von Frey Hairs (0.4, 0.6, 1.4, 2, 4, 6, 8, 10, and 15g) according to the up-down method described by Dixon (19). Each von Frey hair was held for about 1-2 s, with 10-min intervals between each stimulation. Testing was initiated with the 2.0 g hair.

The positive response was defined as a withdrawal of hind paw upon the stimulus. Whenever a positive response appeared, the next lower gram was applied, and whenever a negative response occurred, the next higher gram was applied. The testing consisted of five more stimuli after the first change in response occurred, and the pattern of response was converted to a 50% von Frey threshold using the method described by Dixon and based on our previous literature (20, 21). All data are presented as mean ± SE.

### Sample Collection

To investigate DEPs in three groups, all rats were sacrificed under low dose isoflurane (0.5-1%) anesthesia, six weeks after STZ injection. The L_4-5_ spinal cords were rapidly dissected and immediately placed in -80°C refrigerator for the further proteomics detection.

### Proteomics Experiment and Proteomics Data Analysis

#### Protein Extraction and Trypsin Digestion

The L_4-5_ spinal cord tissue samples were removed from -80°C freezer, and an appropriate amount of sample was added to a pre-chilled mortar with liquid nitrogen and ground to powder. Proteins were then extracted from samples in lysis buffer (8 M urea, 1% protease inhibitor) using a high-intensity ultrasonic processor. Remaining debris was removed by centrifugation at 12,000g for 10min at 4°C. The protein concentration of the supernatant was detected with BAC kits.

For trypsin digestion, protein solution was reduced with dithiothreitol (final concentration, 5mM) for 30 min at 56°C, then incubated with iodoacetamide (final concentration, 11mM) for 15min at 37°C in darkness. Then, the protein samples were diluted with mM triethylamonium bicarbonate (TEAB), until the urea concentration was < 2 M. Finally, trypsin was added at a mass ratio (trypsin: protein) of 1:50 for the first digestion at 37°C overnight, then of 1:100 for the second digestion (4h, 37°C).

#### TMT Labeling

After trypsin digestion, peptides were desalted and vacuum-dried with the strata X C18 SPE column. Peptides were reconstituted in 0.5M TEAB and marked according to the TMT kit protocols.

#### LC-MS/MS Analysis

Separated peptides were subjected to sodium/iodide symporter sources. Tandem mass spectrometry (MS/MS) was performed using a Q ExactiveTM Plus (Thermo) instrument, coupled online to an ultra-performance liquid chromatography system.

Electrospray voltage was 2.0 kV, and intact peptides were detected in the Orbitrap at 70,000 mass resolution. The primary MS scan range was 350–1600 m/z. Collected data were processed using a data-dependent scanning program (DDA). Automatic gain control was set at 5E4, signal threshold as 5000 ions/s, maximum time 200 s, and dynamic exclusion time of the tandem mass scan 15 s, to avoid repeated scanning of precursor ions.

#### Database Search

MS/MS data were analyzed using the max quant search engine (v.1.5.2.8), with parameters as follows: Rat_Uniprot was first screened, then a reverse library added to calculate the false positive ratio (FPR); Trypsin/P was specified as the cleavage enzyme, allowing up to two missing cleavages; minimum peptide length was seven amino acid residues; maximum number of peptide modifications was five; mass tolerance for the primary precursor ions search was 20 ppm and 5 ppm for the main search; mass tolerance for fragment ions was 0.02 Da; the quantitative method was set to TMT-10plex; and the FPR for peptide-spectrum match identification was 1%.

#### Bioinformatics Annotation

Gene Ontology (GO) annotations were derived from the UniProt-GOA database (http://www.ebi.ac.uk/GOA/). InterProScansoft was used to elucidate the GO categories: molecular function, cellular component, and biological process. KEGG online service tools were used to annotate protein descriptions, which were matched into corresponding pathways using KEGG mapper. Wolfpsort (https://wolfpsort.hgc.jp/) was used to investigate subcellular localization. KEGG database pathway enrichment analysis was conducted using a two-tailed Fisher’s exact test. Pathways were classified according to the KEGG website.

Further cluster analysis of function enrichment was conducted to explore potential connections and differences in specific functions. First, all categories were collected after enrichment, according to their P values, then filtered for those categories with P value < 0.05. This filtered P value matrix was transformed using the function x = −log10 (P value). Finally, these x values were z-transformed for each functional category. Cluster membership was visualized using a heat map.

#### Protein-Protein Interaction Network

Numbers or sequences of DEPs were compared using the STRING (v.10.5) protein network interaction database. An interaction relationship was extracted according to a confidence score > 0.7 (high confidence).

### Statistical Analysis

All data are presented as the mean ± standard deviation (SD). The three groups were analyzed by the Analysis Of Variance (ANOVA) and *post hoc* Tukey test in the SPSS 21.0 software (SPSS, Armonk, NY, USA). Graphs were generated by using GraphPad Prism 7.0 software.

## Results

### Electroacupuncture Significantly Reduced Mechanical Hypersensitivity and Fasting Blood Glucose Levels During the Development of DPN.

As shown in the flow chart of the experiment and acupoint diagram (**Figure 1A**), DPN model of rats and EA intervention were established and fasting blood glucose and behavioral tests conducted before and after STZ injection and EA intervention. Compared with the control group, body weight and mechanical pain threshold were significantly reduced while fasting blood glucose was dramatically increased at 2 weeks after STZ injection(**P<0.01).

However, compared with the DPN model group, the body weight and mechanical pain threshold were obviously upgraded, while the fasting blood glucose was soothingly downgraded after four weeks of EA treatment(##*P*<0.01) (**Figures 1B–D**).

### Quantitative of Differentially Expressed Proteins

A total 5744 proteins were identified from spinal dorsal horn samples from the three experimental groups, and 5393 proteins were quantified after proteomic analysis. Molecules with expression ratios showing a > 1.2-fold change and p-values < 0.05 were considered DEPs.

**Figure 2:**
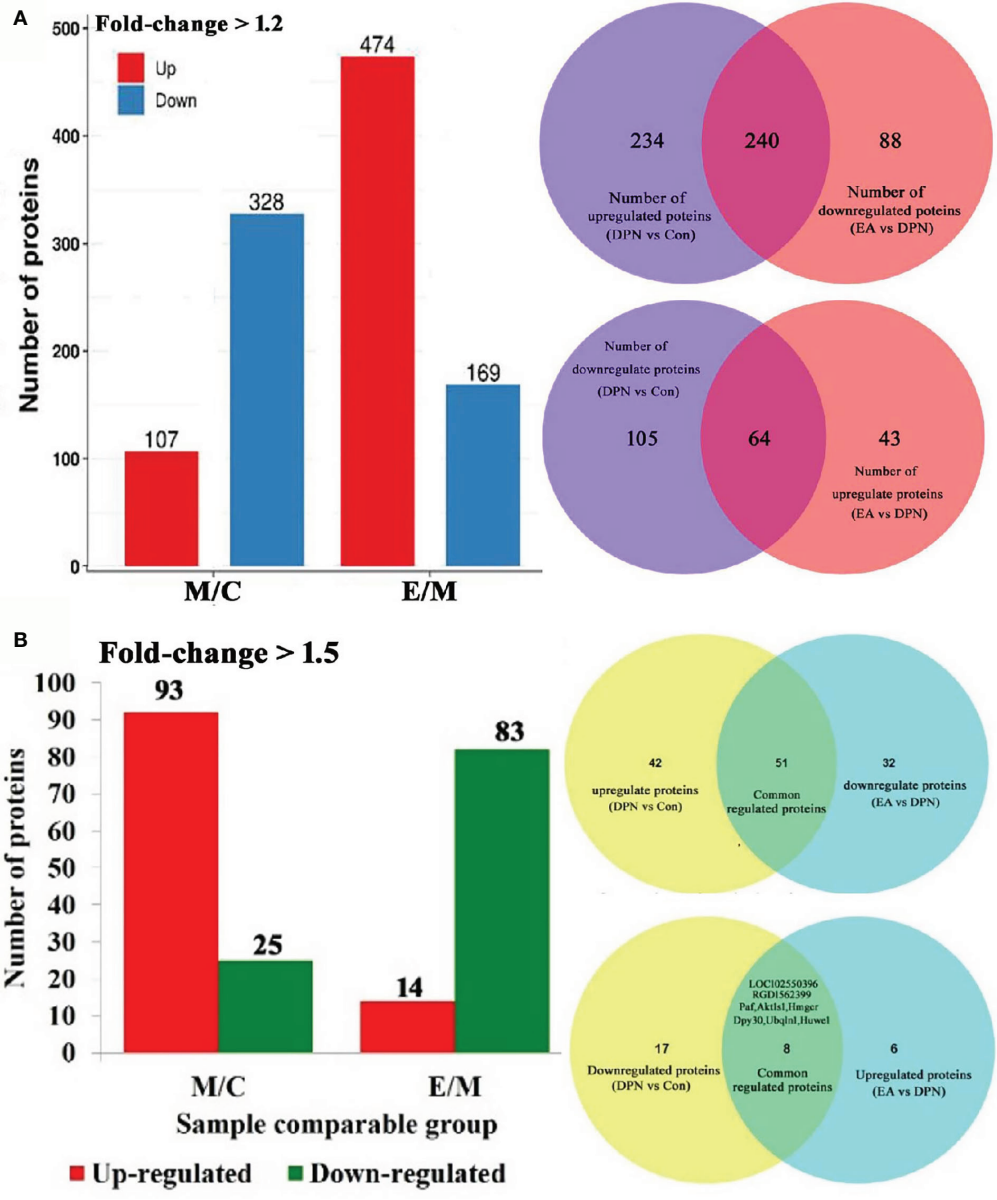
Quantitative of differentially expressed proteins in three groups. **(A)** Number of differential protein set at 1.2-fold (M/C, DPN group compared with control group E/M; EA group compared with DPN group). 240 common positive proteins when DPN up-regulated and EA down-regulated set at 1.5-fold. 64 common positive proteins when DPN down-regulated and EA up-regulated set at 1.5-fold. **(B)** Number of differential protein set at 1.5-fold (M/C, DPN group compared with control group E/M; EA group compared with DPN group). 51 common positive proteins when DPN up-regulated and EA down-regulated set at 1.5-fold. 8 common positive proteins when DPN down-regulated and EA up-regulated set at 1.5-fold.

Compared with the control group, levels of 435 proteins were significantly changed (328 down- and 107 up-regulated) in DPN rats and 643 proteins changed in EA rats (p < 0.05 and fold-change > 1.5) (**Figure 2A**). Further analysis showed that 118 proteins were significantly changed (25 down- and 93 up-regulated) in DPN rats while 14 proteins were up-regulated and 83 proteins down-regulated after EA treatment (p < 0.05 and fold-change > 1.5) (**Figure 2B**). There were 51 common positive proteins when DPN up-regulated and EA down-regulated (p < 0.05 and fold-change > 1.5), while 8 common positive proteins were found which DPN down-regulated and EA-upregulated (p < 0.05 and fold-change > 1.5). There were total 59 common changed proteins shown in the **Table 1** (p < 0.05 and fold-change > 1.5).

**Table 1:** The common significant differences of EA-regulated and DPN-associated proteins (p < 0.05 and changes >1.5-fold).

Protein accession	Protein description	Gene name	DPN/CON Ratio	P value	EA/DPN Ratio	P value
F7F9X9	—	Slc26a8	14.35	2.28E-06	0.076	1.61E-05
D3ZUF8	—	Vrk2	4.899	3.56E-05	0.216	0.000162
F7EUK4	T-kininogen 2	Kng1l1	4.212	2.17E-05	0.449	4.18E-05
Q5YIR9	—	Clec4a1	3.793	1.69E-08	0.225	0.001378
P17712	Glucokinase	Gck	3.655	6.97E-07	0.311	0.003456
P01048	T-kininogen 1	Map1	3.457	3.28E-06	0.528	4.31E-05
A0A0H2UHM3	Haptoglobin	Hp	2.313	0.035243	0.451	0.000338
M0RAV0	—	—	2.242	0.000317	0.646	0.001018
F1M5A4	Katanin p60 ATPase-containing subunit A-like 2	Katnal2	2.238	1.69E-05	0.48	2.22E-05
M0R4Z4	—	LOC682352	2.207	4.27E-05	0.636	0.000698
P20767	Ig lambda-2 chain C region	—	1.995	0.013403	0.642	5.97E-05
G3V9P3	—	MGC112715	1.95	2.46E-05	0.499	3.88E-05
D3ZNA5	Class I histocompatibility antigen, Non-RT1.A alpha-1 chain	LOC686860	1.878	4.33E-05	0.519	4.1E-05
B1WBQ8	Glyceraldehyde-3-phosphate dehydrogenase	Gapdhs	1.848	0.007075	0.626	0.001681
Q7TQ70	Fibrinogen alpha chain	Fga	1.823	1.17E-06	0.647	3.94E-06
D3ZV51	Olfactory receptor	LOC100909611	1.822	0.010757	0.427	0.000603
D4A6F2	—	Lynx1	1.822	0.000436	0.605	0.000517
A0A0G2KB63	Prohibitin-2	Phb2	1.821	3.07E-06	0.55	4.52E-06
P02680	Fibrinogen gamma chain	Fgg	1.796	4.31E-05	0.664	0.000184
P14480	Fibrinogen beta chain	Fgb	1.789	1.58E-05	0.644	1.64E-05
P67779	Prohibitin	Phb	1.751	3.53E-05	0.555	0.000384
F1M4J0	—	Rictor	1.748	0.00012	0.59	9.54E-05
A0A096MJ90	—	Ly6h	1.713	8.48E-05	0.529	6.06E-05
Q7TPI6	—	rCG_62991	1.69	0.001398	0.643	0.010803
Q66HL0	5-nucleotidase	Nt5e	1.689	0.003575	0.644	0.008803
P11951	Cytochrome c oxidase subunit 6C-2	Cox6c2	1.647	1.33E-06	0.495	4.16E-05
G3V851	—	Slc17a6	1.64	0.001939	0.608	9.99E-05
P80431	Cytochrome c oxidase subunit 7B, mitochondrial	Cox7b	1.634	1.48E-06	0.537	8.1E-07
B0BNE6	—	Ndufs8	1.62	0.000135	0.647	0.000305
P01836	Ig kappa chain C region, A allele	—	1.597	0.002182	0.362	0.000221
P10888	Cytochrome c oxidase subunit 4 isoform 1, mitochondrial	Cox4i1	1.574	4.74E-06	0.573	1.62E-06
P11661	NADH-ubiquinone oxidoreductase chain 5	Mtnd5	1.572	0.001844	0.597	5.64E-05
P80432	Cytochrome c oxidase subunit 7C, mitochondrial	Cox7c	1.562	0.000196	0.5	0.000103
D3ZLT1	—	Ndufb7	1.559	5.66E-05	0.585	8.2E-05
D4A565	—	Ndufb5	1.557	2.11E-05	0.582	4.47E-06
F1LXA0	—	Ndufa12	1.555	1.88E-05	0.625	3.16E-06
D3ZYX8	—	Cox7a2l	1.547	0.000164	0.631	0.000143
F1LTP5	Coiled-coil domain-containing protein 63	Ccdc63	1.546	8.42E-05	0.622	0.00018
A0A0G2K7D4	Dynein heavy chain 12, axonemal	Dnah12	1.545	2.08E-05	0.611	0.000599
Q5M7T6	—	Atp6v0d1	1.545	3.57E-05	0.58	4.5E-05
D4ACP8	—	Serac1	1.542	0.000463	0.664	0.00038
D4A463	—	Tpgs1	1.535	0.024738	0.579	0.006541
D3ZG43	—	Ndufs3	1.521	1.75E-05	0.641	1.69E-06
A0A2U3UXS5	—	Igsf21	1.519	0.006782	0.583	0.00152
A0A0H2UHV2	Mediator of RNA polymerase II transcription subunit 23	Med23	1.514	0.000217	0.666	0.000244
F1M0G0	—	Rgs17	1.51	0.006183	0.579	0.001856
P12075	Cytochrome c oxidase subunit 5B, mitochondrial	Cox5b	1.507	3.79E-05	0.587	4.56E-06
F1M9G7	CREB-binding protein	Crebbp	1.506	0.00094	0.623	6.26E-05
B0BN66	—	Sapcd2	1.505	0.005243	0.635	0.003216
B5DEL8	—	LOC100363268	1.504	0.000462	0.663	0.000245
F1LPG5	—	LOC688963	1.5	6.33E-05	0.637	0.000138
**Protein accession**	**Protein description**	**Gene name**	**DPN/CON Ratio**	**P value**	**EA/DPN Ratio**	**P value**
A0A0G2JUM9	—	LOC102550396	0.366	2.4303E-05	2.492	0.0001
D3ZAS1	—	RGD1562399	0.419	3.5258E-05	2.183	0.000136
Q6RIA2	PCNA-associated factor	Paf	0.468	5.7355E-05	1.548	0.001543
D3ZH75	—	Akt1s1	0.525	0.0183551		
P51639	3-hydroxy-3-methylglutaryl-coenzyme A reductase	Hmgcr	0.601	0.0025202	1.573	0.007535
G3V946	—	Dpy30	0.639	0.0141759	1.757	0.005797
A0A140TAI1	—	Ubqln1	0.647	4.0053E-05	1.54	0.000103
P51593	E3 ubiquitin-protein ligase HUWE1	Huwe1	0.665	5.9014E-05	1.543	0.00098

### Molecular Function, Cellular Component, and Biological Process in Control, DPN, and EA Group Rats

GO analysis was used to evaluate the molecular function, cellular component, and biological processes enriched for DEPs.

**Figure 3:**
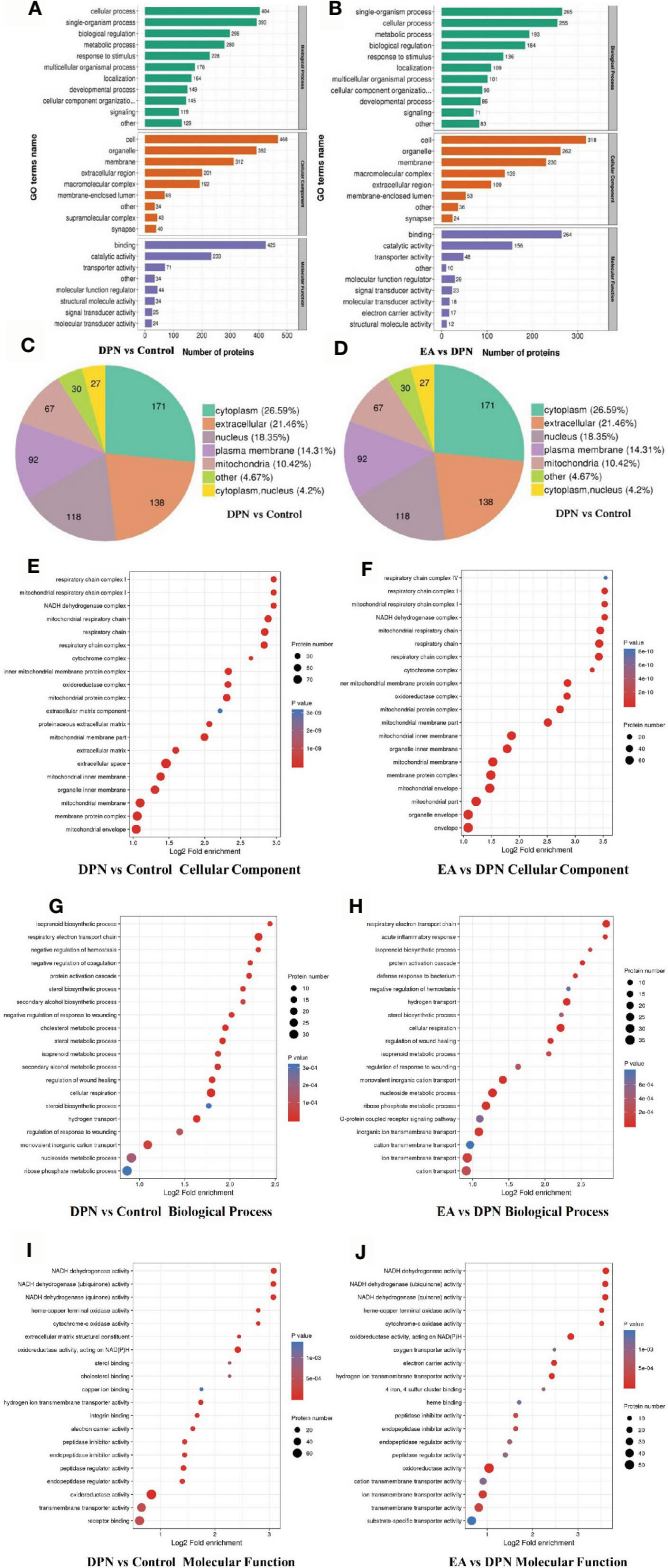
GO annotation analysis showed the potential biomarkers mainly involved in DPN pathology and EA treatment **(A)** GO terms name of molecular function, cellular component, and biological process in DPN-associated proteins **(B)** GO terms name of molecular function, cellular component, and biological process in EA-regulated proteins **(C)** Subcellular structural localization analysis in DPN-associated proteins **(D)** Subcellular structural localization analysis in EA-regulated proteins. **(E–J)** GO annotation summarizing in cellular component, molecular function and biological process in different compared groups.

In terms of biological processes, the top five enriched for DPN-associated proteins were cellular process, single-organism process, biological regulation, metabolic process, and response to stimulus, while the top five enriched for EA-regulated proteins were single-organism process, cellular process, metabolic process, biological regulation, and response to stimulus. Cell component analysis indicated the most enriched for DPN-associated proteins were cell, organelle, membrane, extracellular region, and macromolecular complex, among others, while EA-regulated proteins were mainly involved in the cell, organelle, membrane, macromolecular complex, and extracellular region. Molecular functions analysis suggested that the most common molecular functions of DPN-associated and EA-regulated proteins were binding and catalytic activity (**Figures 3A, B**).

According to subcellular structural localization analysis, the main subcategories (> 10%) in DPN-associated proteins were cytoplasm (26.59%), extracellular (21.46%), nucleus (18.35%), plasma membrane (14.31%), and mitochondria (10.42%). The same analysis of EA-associated proteins also identified the cytoplasm (27.36%), extracellular (18.62%), nucleus (17.47%), plasma membrane (15.4%), and mitochondria (13.56%) subcategories (**Figures 3C, D**).

Last, GO analysis demonstrated that the cellular component, biological process molecular function of DPN and EA treatment were closely related to the mitochondrial respiratory chain and NADH dehydrogenase activity (**Figures 3E–J**).

### Functional Enrichment Analysis With KEGG Pathway and Cluster Analysis of KEGG Pathway Functional Correlation of the DEPs

**Figure 4:**
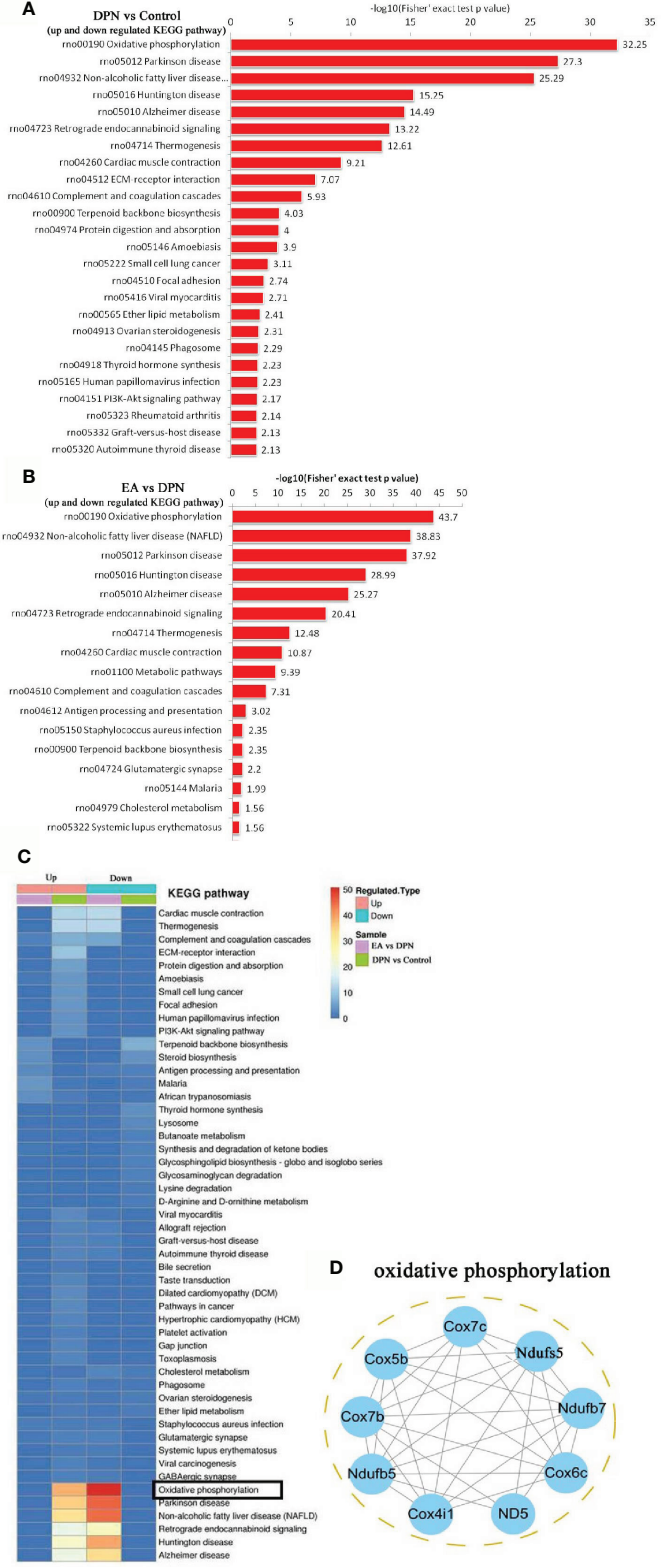
KEGG pathway and related oxidative phosphorylated proteins analysis **(A)** the KEGG pathway enrichment analysis in DPN rats compared with control rats. **(B)** The KEGG pathway enrichment analysis in EA group rats compared with DPN rats. **(C)** The KEGG pathway analysis in different group rats. **(D)** Networks Analysis of major proteins in oxidative phosphorylation.

To further explore pathways significantly enriched for DEPs (DPN *vs*. Control, n = 643; EA *vs*. DPN, n = 435), DEPs were mapped to the KEGG database and enrichment levels calculated (Fisher’s exact test; -log 10[p-value]), where a higher p-value indicates more DEPs enriched in a category. As shown in **Figures 4A, B**, KEGG pathway enrichment analysis demonstrated that DPN-associated and EA-regulated DEPs were mainly enriched in several pathways, particularly oxidative phosphorylation (**Figure 4C**). Further, remarkable changes were observed in ten proteins closely related to oxidative phosphorylation during EA treatment of DPN rats (**Table 2** and **Figure 4D**).

**Table 2:** The prominent changes of oxidative phosphorylated proteins during EA treatment with DPN rat.

Protein accession	String gene	Protein description	DPN vs Control	EA vs DPN
P80432	Cox7c	Cytochrome c oxidase subunit 7C, mitochondria	1.562 up	0.5 down
P12075	Cox5b	Cytochrome c oxidase subunit 5B, mitochondria	1.507 up	0.587 down
P80431	Cox7b	Cytochrome c oxidase subunit 7B, mitochondria	1.634 up	0.537 down
D4A565	Ndufb5	NADH dehydrogenase (Ubiquinone) 1 beta subcomplex 5	1.557 up	0.582 down
P10888	Cox4i1	Cytochrome c oxidase subunit 4 isoform 1, mitochondria	1.574 up	0.573 down
P11661	ND5(Mtnd5)	NADH-ubiquinone oxidoreductase chain 5	1.572 up	0.597 down
P11951	Cox6c2	Cytochrome c oxidase subunit 6C-2	1.647 up	0.495 down
D3ZLT1	Ndufb7	“NADH dehydrogenase (Ubiquinone) 1 beta subcomplex, 7	1.559 up	0.585 down
B5DEL8	Ndufs5	NADH dehydrogenase (Ubiquinone) Fe-S protein 5	1.504 up	0.663 down

## Discussion

In our research, we found that four weeks EA can improve weight loss, blood glucose, and sensitivity to mechanical pain in DPN rats. We generated proteomics data from DPN rat spinal dorsal horn samples treated with repeated EA stimulation. A total of 5393 proteins were quantified using the TMT labeling proteomics method. Of these proteins, 93 were down-regulated, while 25were up-regulated during the development of DPN. After EA stimulation intervention, 14 up-regulated and down-regulated 83 proteins were detected in the spinal dorsal horn. KEGG pathway enrichment analysis suggested that oxidative phosphorylation was a major factor involved in the effects of EA therapy on DPN. The results of this study provide a potentially valuable theoretical underpinning of the mechanisms underlying DPN pathophysiology and the clinical application of acupuncture.

The occurrence of diabetes and its complications are closely related to the mechanism of oxidative phosphorylation *via* the disturbances of mitochondrial ATP, complex IV and reactive oxygen species (ROS) metabolism (22–24). Upregulated expression of complexes I, III, IV, and V was found in the sciatic nerve of diabetic neuropathy rats (11). In addition, mitochondrial complex III of dorsal root ganglia was significantly decreased, while glutathione peroxidase and peroxidase were increased during STZ induced diabetic peripheral neuropathy (13).

Our results indicate that cytochrome c oxidase (Cox, Complex IV) factors, including Cox4i1, Cox5b, Cox6c2, Cox7b, and Cox7c, were significantly upregulated in spinal dorsal horn during DPN, as were NADH-ubiquinone oxidoreductase (Complex I) factors, including Ndufb5, Ndufb7, andNdufs5. Further, treatment with EA could decrease the up-regulation of Cox and NADH oxidoreductase. As we know, abnormal function of mitochondrial Cox and NADH oxidoreductase could induce ROS overproduction, caspase 3 activation cell apoptosis (25, 26). Spinal ROS overproduction and activated caspase-3 induced by Cox play a critical role in microglial activation and neuronal apoptosis in several neuropathic pain models included DPN (27, 28). Blocking NADPH oxidase, scavenging ROS, and reducing oxidative phosphorylation by various pharmacotherapy could prevent the induction of spinal long-term potentiation and relieve the persistent pain (29–31).

In addition, EA can also inhibit oxidative phosphorylation and ROS production through different pathways in neuropathic pain and other chronic diseases. For example,

Neuronal ATP receptor P2X7 activation leads to ROS production and subsequent nociceptive pain (32, 33). EA can improve neuropathic pain by inhibiting oxidative phosphorylation, reducing ATP production, and suppressing central sensitization in the spinal dorsal horn (34). In middle cerebral artery occlusion rats, EA treatment could significantly improve the respiratory control ratio, and increase the activities of NADH dehydrogenase and Cox in the perifocal penumbra zone (35). In the state of ischemia reperfusion injury, EA can inhibit cardiac Cox and indirectly inhibit the activation of caspase 3 (36). However, EA can alleviate bupivacaine-induced myocardial injury by upregulating enzymatic activity of Cox (37). In a word, EA can biaxially regulate oxidative phosphorylation of Cox and NADH dehydrogenase with bidirectional properties in different diseases and tissues.

According to the above research reports, combined with our results, we speculated that EA can reduce diabetic neuropathic pain by downregulating Cox and NADH dehydrogenase in the spinal cord, which may mainly be through the following ways: (1) inhibiting the spinal ATP release and P2X7 activation, reducing the spinal neuronal abnormal excitability and microglial activation; (2) reducing spinal ROS production and oxidative phosphorylation injury, inhibiting cell injury and even apoptosis. In the next experiment, we will further explore the mechanism of EA regulating ROS and oxidative phosphorylation in DPN.

## Conclusion

In conclusion, we used a TMT labeling approach coupled with liquid chromatography with tandem mass spectrometry to show that DPN and EA stimulation drive significant changes in spinal proteins, particularly those involved in oxidative phosphorylation. Our results highlight potential candidate protein biomarkers for DPN diagnosis and treatment.

## Data Availability Statement

The datasets presented in this study can be found in online repositories. The names of the repository/repositories and accession number(s) can be found in the article/supplementary material.

## Ethics Statement

The animal study was reviewed and approved by Fujian University of Traditional Chinese Medicine Animal Ethics Committee.

## Author Contributions

XY and XC made an equal contribution to this research. ZW and JT are the co-corresponding authors and they completed the project design, data analysis, and drafted part of the manuscript. All authors contributed to the article and approved the submitted version.

## Funding

This study was jointly supported by the National Natural Science Foundation of China (81774385, 81704149), the Science and technology platform construction project of Fujian science and Technology Department(Grant No.2018Y2002), the Key Project of “Traditional Chinese Medicine modernization research” in the National Key Research and Development plan (2019YFC1710301).

## Conflict of Interest

The authors declare that the research was conducted in the absence of any commercial or financial relationships that could be construed as a potential conflict of interest.
